# Whole Blackcurrant (*Ribes nigrum*) Alleviates High‐Fat Diet Induced‐Obesity and Colonic Inflammation by Modulating the Gut Microbiota in Mice

**DOI:** 10.1002/mnfr.70462

**Published:** 2026-04-15

**Authors:** Hye‐Jung Moon, Youn‐Soo Cha, Kyung‐Ah Kim

**Affiliations:** ^1^ Department of Food Science and Human Nutrition Jeonbuk National University Jeonju‐si, Jeonbuk‐do Republic of Korea; ^2^ K‐Food Research Center Jeonbuk National University Jeonju‐si, Jeonbuk‐do Republic of Korea; ^3^ Department of Food and Nutrition Chungnam National University Daejeon, Yuseong‐gu Republic of Korea

**Keywords:** blackcurrant, colonic inflammation, dysbiosis, gut microbiota, obesity, whole‐foods

## Abstract

Obesity, a chronic low‐grade inflammatory state, remains a major global health challenge. Recently, the gut microbiota and intestinal immune system have been identified as playing key roles in obesity, leading to their proposal as novel targets for preventing obesity and its related complications. The present study aimed to determine whether whole blackcurrant (BC) could improve colonic inflammation and obesity by regulating the gut microbiota. The 6‐week‐old male C57BL/6J mice were divided into three groups and fed either a normal diet, a high‐fat diet (HFD), or an HFD containing 6% whole BC for 16 weeks. As a result, whole BC showed beneficial effects on body weight, abdominal fat volume, and lipid profiles in the liver and serum. Whole BC ameliorated mucosal barrier damage induced by an HFD, thereby reducing LPS translocation into the bloodstream. Furthermore, it decreased intestinal and systemic inflammation by attenuating the excessive secretion of pro‐inflammatory cytokines through the suppression of NF‐κB signaling pathway overactivation in the colon. Whole BC reversed gut microbiota dysbiosis caused by an HFD. Therefore, the whole BC provides benefits against obesity by improving the mucosal barrier and colonic inflammation through the regulation of the gut microbiota altered by an HFD, thereby alleviating systemic inflammation.

## Graphical abstract

Obesity caused by a high‐fat diet (HFD) is known to result from interactions between the gut microbiota, intestinal barrier function, and excessive inflammatory responses in the colon. In this study, we found that dietary intake of whole blackcurrants prevented HFD‐induced obesity by restoring gut microbiota dysbiosis, upregulating intestinal barrier function‐related gene expression, and inhibiting overactivated NF‐κB signaling pathway, leading to attenuated colonic and systemic inflammation.

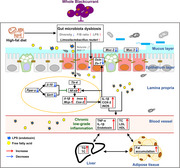

**Abbreviations**
ACN: Anthocyanins
ASV: Amplicon sequence variant
BC: Blackcurrant
BCA: Bicinchoninic acid
CLDN1: Claudin1
Col1a1: Collagen type 1
COX‐2: Cyclooxygenase‐2
MCP‐1: Monocyte chemoattractant protein‐1
CTGF: Connective tissue growth factor
ECL: Enhanced chemiluminescence
EER: Energy efficiency ratio
F/B ratio: The ratio of *Firmicutes* to *Bacteroidetes*
FER: Food efficiency ratio
GAPDH: Glyceraldehyde 3‐phosphate dehydrogenase
HFD: High‐fat diet
HRP: Horseradish peroxidase
LDA: Linear discriminant analysis
LEfSe: LDA effect size
Micro‐CT: Micro‐computed tomography
MUC: mucin
iNOS: Inducible nitric oxide synthase
NASH: Nonalcoholic steatohepatitis
NF‐κB: Nuclear factor kappa‐light‐chain‐enhancer of activated B cells
Ocln: Occludin
Zo‐1: Zonula occludens‐1
PC: Principal component
PCoA: Principal coordinates analysis
PPAR: Peroxisome proliferator‐activated receptor
PVDF: Polyvinylidene difluoride
SCFAs: Short‐chain fatty acids
SFAs: Saturated fatty acids
TBST: Tris‐buffered saline with tween‐20
TC: Total cholesterol
TNF‐α: Tumor necrosis factor‐α
RIPA: Radioimmunoprecipitation assay
H&E: Hematoxylin and eosin
TG: Triglycerides
TJ: Tight junction proteins;
TLR‐4: Toll‐like receptor‐4

## 1 Introduction

Obesity is known to result from a combination of genetic, environmental, and social factors with diet being a key modifiable environmental factor [1, 2, 3]. In particular, high‐fat intake leads to chronic metabolic inflammation by causing excessive lipid accumulation in adipose tissue as well as metabolic dysfunction by inducing ectopic fat deposition in other organs [4]. Thus, obesity has been linked to a chronic low‐grade systemic inflammation, contributing to the development of type 2 diabetes, cardiovascular disease, nonalcoholic steatohepatitis (NASH), and other conditions [4]. Although the pathophysiological mechanisms underlying obesity have not yet been fully identified, recent studies have focused on the role of the gut microbiota in obesity and related diseases [5]. Environmental factors such as diet can cause dysbiosis of the gut microbiota, affecting the intestinal immune system and leading to endotoxemia, which can promote obesity and its associated complications [5, 6]. Obesity prevalence has nearly tripled worldwide in the last 50 years and continues to be a major global health challenge [1], requiring new prevention and management strategies.

A high‐fat diet (HFD) is a primary contributor to obesity. Previous studies have shown that an HFD with saturated fatty acids (SFAs) causes dysbiosis of commensal bacteria in the intestinal lumen, increases endotoxin (such as LPS), activates the toll‐like receptor‐4 (TLR‐4)/nuclear factor kappa‐light‐chain‐enhancer of activated B cells (NF‐κB) signaling pathway in the intestinal barrier, and triggers intestinal inflammatory responses, ultimately leading to increased intestinal permeability [3, 6]. Increased intestinal permeability allows gut microbiota, toxins, and inflammatory cytokines to pass through the intestinal barrier and enter the circulatory system, thereby increasing metabolic inflammation and accelerating obesity [3, 6].

This evidence highlights the complex pathophysiological link between obesity and intestinal inflammation [7, 8]. Notably, an HFD‐induced intestinal inflammation was ameliorated by the gut anti‐inflammatory agent 5‐aminosalicylic acid, which reduced inflammation in visceral adipose tissue and facilitated barrier recovery, attracting research attention as a target for obesity treatment [9]. Given that gut dysbiosis and chronic intestinal inflammation have been proposed as key contributors to obesity, therapeutic strategies targeting these factors are being extensively investigated, including dietary interventions and natural product‐based approaches [2, 10].

Recently, various plants including fruits, legumes, vegetables, grains, and teas have been reported to exhibit anti‐obesity effects by regulating the gut microbiota [2]. Among these, anthocyanins (ACN), which are abundant in berries, have been shown to restore the gut microbiota imbalance induced by HFDs and inhibit the inflammatory response, consequently preventing obesity [11, 12]. This evidence suggests that berries have potential as a dietary intervention to prevent obesity by modulating the gut microbiota.

Blackcurrant (BC), scientifically known as *Ribes nigrum* is a small blackberry that is mainly consumed as raw fruit, beverages, powder, syrup, and jams [13]. The primary ACNs in BC include cyanidin glycosides (cyanidin 3‐*O*‐glucoside and cyanidin 3‐*O*‐rutinoside) and delphinidin glycosides (delphinidin 3‐*O*‐glucoside and delphinidin 3‐*O*‐rutinoside) [13], and BC has the highest content of polyphenols, flavonoids, and monomeric ACNs among small dark fruits [14]. ACNs in BC ameliorate oxidative stress, inflammation, diabetes‐related metabolic disorders, and muscle damage [14, 15, 16].

Moreover, ACNs in BC have been shown to prevent obesity by regulating gut microbiota and lipid metabolism [17]. Thus, BC represents a promising dietary intervention for the prevention of both obesity and intestinal inflammation. Consumption of whole foods, such as fruits and vegetables, is known to reduce the risk of obesity and inflammatory bowel disease by modulating gut microbiota [10]. Furthermore, previous studies suggest that ACNs may be more effective when consumed as whole foods than as isolated extracts, potentially owing to food matrix‐mediated improvements in stability and bioavailability [18, 19].

Blackcurrant (BC) is routinely processed and consumed in various products in the whole form [13]. However, most studies have focused on BC extracts, and studies investigating whether the intake of whole BC has beneficial effects are currently limited. Some studies have shown that the whole form of BC inhibits macrophage infiltration into adipose tissue and NASH in diet‐induced obese mice, as well as providing a protective effect against chemically induced colitis [20, 21, 22]. Despite these findings, a comprehensive understanding of how whole BC intake impacts the interplay among gut microbiota, colonic inflammation, and obesity‐related metabolic profiles remains limited. The HFD‐induced obesity model is widely used to examine the pathological interactions underlying obesity and to mimic human obesity and its related disorders [3, 23].

Thus, the intake of whole BC is expected to have beneficial effects on gut microbiota, intestinal inflammatory response, and obesity. Therefore, this study aimed to investigate whether dietary intake of freeze‐dried whole BC could prevent obesity by modulating the gut microbiota and alleviating inflammation in the colon, where the gut microbiota is the most abundant, and furthermore, to evaluate its potential as a dietary strategy for obesity prevention and management.

## 2 Experimental Section

### 2.1 Animals

The 6‐week‐old male C57BL/6J mice were purchased from Central Laboratory Animal Inc. (Seoul, Republic of Korea). Mice were placed in a controlled environment with a 12 h light/dark cycle at a temperature of 22 ± 2°C and relative humidity of 50 ± 5%. During the acclimatization period of 5 days, the mice were given a normal chow diet.

All animal protocols were approved by the Institutional Animal Care and Use Committee of Chungnam National University (approval number: 202103A‐CNU‐033) and performed in compliance with the Animal Protection Act and Laboratory Animal Act of the Republic of Korea.

### 2.2 Experimental Design

After the adaptation period, mice were divided into 3 groups (*n* = 8 per group, total *n* = 24) and fed the following experimental diet ad libitum for 16 weeks. i) NF group, a normal diet with 10% energy from fat; ii) HF group, an HFD with 60% energy from fat, iii) HFB6 group, an HFD with 60% energy from fat added with 6% freeze‐dried whole BC powder. Freeze‐dried whole BC powder is a commercially available product (Sujon Berries, Nelson, New Zealand). The normal diet was prepared based on the published formulation of D12450J (Research Diets, Inc., New Brunswick, NJ, USA). Freeze‐dried whole BC powder was incorporated into the HFD at 6% (w/w; 60 g/kg diet), a concentration previously reported to prevent nonalcoholic steatohepatitis in obesity‐induced murine models [20]. Based on previously reported total ACN content for the BC powder (23.1 mg ACN/g powder) [24], the ACN concentration in the experimental diet was estimated to be 1.39 mg/g diet. Animals had ad libitum access to diet and water. Considering the mean food intake (2.5 g/mouse/day) observed in the BC‐ supplemented group, the daily intake of BC powder was estimated at 0.15 g/mouse. This corresponds to a human equivalent dose of 20.7 g/day for a 60 kg adult, calculated using the body surface area‐based conversion formula [25]. The detailed composition of the diet is shown in Table S1.

The food intake was measured three times a week, and the body weight was monitored once a week. The food efficiency ratio (FER) or energy efficiency ratio (EER) was calculated as a percentage (%) by dividing the body weight gain by the dietary intake or energy intake, respectively. The volume of abdominal fat was evaluated by Micro‐Computed Tomography (Micro‐CT) at week 15. Feces were collected one day before sacrifice to analyze gut microbiota.

At the end of week 16, the mice were fasted for 12 h, anesthetized with isoflurane, and whole blood was collected from the inferior vena cava. After sacrifice, the liver and epididymal fat were collected and weighed. The colon was removed and its length was measured. For histological analysis, a 0.5 cm segment from the middle portion of the colon was fixed in 4% formalin solution. For RNA extraction, ELISA, and western blot analyses, colon samples were prepared to include the entire colon (proximal, middle, and distal portions). Colon samples, liver, and epididymal fat were rapidly frozen in liquid nitrogen and stored at −70°C until analysis. To obtain serum, blood was allowed to stand at room temperature for 2 h and then centrifuged at 4°C and 1500 ×g for 15 min.

### 2.3 Micro‐CT Image

Abdominal fat images of mice were obtained by scanning the lumbar vertebrae (L1–L5) using Micro‐CT (SKYSCAN 1076, SkyScan, Aartselaar, Belgium) installed in the Center for University‐wide Research Facilities (CURF) at Jeonbuk National University. The volume of abdominal fat was calculated using regions of interest (ROI) by CTAn software (SkyScan).

### 2.4 Biochemical Parameters

Lipid profiles such as triglycerides (TG), total cholesterol (TC), and HDL‐cholesterol were performed using commercial enzymatic assay kits (Asan Pharm. Co., Ltd., Hwaseong, Republic of Korea) according to the manufacturer's instructions. In serum, TG, TC, and HDL were tested, and LDL‐cholesterol was calculated using the Friedewald equation [26]: TC—(HDL + (TG/5)). The TG and TC in liver tissue were analyzed with the assay kit described above after total lipids were extracted with a solvent (2:1) mixed with chloroform and methanol [27].

The levels of tumor necrosis factor‐α (TNF‐α), IL‐1β, and IL‐6 in the serum and colon tissue were quantified according to the manufacturer's protocol by using an ELISA assay kit (TNF‐α, Invitrogen, Vienna, Austria; IL‐1β and IL‐6, R&D Systems, Minneapolis, MN, USA). Colon tissue was homogenized on ice in a radioimmunoprecipitation assay (RIPA) buffer (Thermo Scientific) containing 1% phosphatase inhibitor cocktail (#04906837001, Roche Diagnostics, Mannheim, Germany) and 1% protease inhibitor cocktail (#04693159001, Roche Diagnostics), and then centrifuged (13,000 x g, 10 min, 4°C) and the supernatant was collected and used for ELISA assay. Endotoxin quantification in serum was determined using a commercially available Chromogenic Endotoxin Quant kit (#A39552, Thermo Scientific, Rockford, IL, USA).

### 2.5 Histological Analysis

The fixed colon tissue was embedded in paraffin, and then 4 µm sections were prepared and hematoxylin and eosin (H&E) staining and Masson's trichrome staining were performed. Stained glass slides were observed using a light microscope (DM2500, Leica Microsystems, Wetzlar, Germany). The histological score for colonic inflammation was evaluated by observing the architecture, length, and abscesses of the crypt, goblet cell loss, inflammatory cell infiltration, and lamina propria neutrophils per high power field [28]. Mucus and muscle thicknesses were measured using ImageJ software (National Institutes of Health, Bethesda, MD, USA). All parameters were quantified in five randomly selected fields per stained colon section.

### 2.6 Real‐Time PCR Analysis

Total RNA from colon tissue was extracted using the RNeasy Mini kit (#74106, Qiagen, Hilden, Germany). The extracted RNA was evaluated for purity using a BioDrop Duo spectrophotometer (Biochrom Ltd., Holliston, MA, USA). Reverse transcription was performed by RT Master Mix (#RR036A, Takara Bio Inc., Shiga, Japan). Total RNA (1000 ng) was reverse‐transcribed into cDNA using an RT Master Mix (#RR036A, Takara Bio Inc., Shiga, Japan) according to the manufacturer's instructions. Expression of the target gene was confirmed by amplifying with the 7500 Real‐time PCR system (#4345241, Applied Biosystems, Foster City, CA, USA) using the SYBR Green Master mix (#QPK‐201, Toyobo Co., Ltd., Osaka, Japan) and the target prime (Table S2). Expression of the target mRNA was normalized by glyceraldehyde 3‐phosphate dehydrogenase (GAPDH) and expressed as a fold change relative to the NF group as a control.

### 2.7 Western Blot Analysis

Protein extract from the colon tissue was obtained in accordance with the method of pre‐processing the colon tissue for ELISA analysis. The protein concentration of the colonic lysate was detected using the bicinchoninic acid (BCA) protein assay kit (#23227, Thermo Scientific). The lysates were denatured by adding 5X loading buffer containing 2‐mercaptoethanol (#S0424, Curebio, Seoul, Republic of Korea) and heating at 95°C for 5 min, and separated by 8%–10% SDS‐PAGE. The separated protein was transferred to 0.2 µm polyvinylidene difluoride (PVDF) membranes (#1620177, Biorad Laboratories Inc., USA). The membrane was blocked with 5% skim milk in Tris‐buffered saline with tween‐20 (TBST) buffer for 1 h, washed with TBST buffer, and incubated with primary antibodies overnight at 4°C. Then, it was washed again with TBST buffer and incubated with the corresponding horseradish peroxidase (HRP)‐conjugated secondary antibody for 1 h. The primary and secondary antibodies used in this study are listed in Table S3. The specific signals were visualized using enhanced chemiluminescence (ECL) reagent and ChemiDoc system (ATTO Lumino Graph II, ATTO, Tokyo, Japan).

### 2.8 Gut Microbiota Analysis

The collected feces were commercially commissioned to Macrogen Inc. (Seoul, Republic of Korea) for analysis of gut microbiota. Bacterial DNA was extracted from feces, and the bacterial 16S rRNA V3‐V4 region was amplified using 341F and 805R primers. The library of 16S rRNA was produced with Nextera XT index V2 kit (Illumina Inc., San Diego CA, USA). The prepared amplicon was sequenced using Illumina MiSeq (Illumina, Inc.). Raw fastq files were processed with the QIIME2 program to obtain the feature table of the amplicon sequence variant (ASV). Based on ASVs, it was used to analyze the structure of the taxonomic and diversity of the gut microbiota. Alpha‐diversity (Chao1, Shannon, and Gini‐Simpson index) and beta‐diversity (principal coordinates analysis (PCoA)) were analyzed with QIIME 2. The taxonomic assignment of gut microbiota was referred to the NCBI 16S BLAST database (https://www.ncbi.nlm.nih.gov/). Relative abundances of phylum, genus, and species were presented as bar graphs. The linear discriminant analysis (LDA) effect size (LEfSe) was expressed using the galaxy tool (https://huttenhower.sph.harvard.edu/galaxy/) in order to find microbes that are likely to differ between three groups.

### 2.9 Statistical Analysis

All results were expressed as mean ± SD. Significant differences between groups were evaluated as a one‐way analysis of variance (ANOVA), followed by Tukey's post hoc tests. A *p*‐value < 0.05 was considered statistically significant, and significant differences were indicated by different superscript letters in the values. The results of gut microbiota analysis were expressed as box‐and‐whisker plots, which include the median, quartiles, and the minimum and maximum values. The correlation analysis between the gut microbial community and selected parameters was determine using Spearman's correlation analysis. Statistical analysis was performed using SPSS 18.0 (SPSS Inc., Chicago, IL, USA) and visualization was carried out utilizing GraphPad Prism 8 (GraphPad Software, San Diego, CA, USA).

## 3 Results

### 3.1 Freeze‐Dried Whole BC Prevents Body Weight Gain and Fat Accumulation Induced by an HFD

**FIGURE 1:**
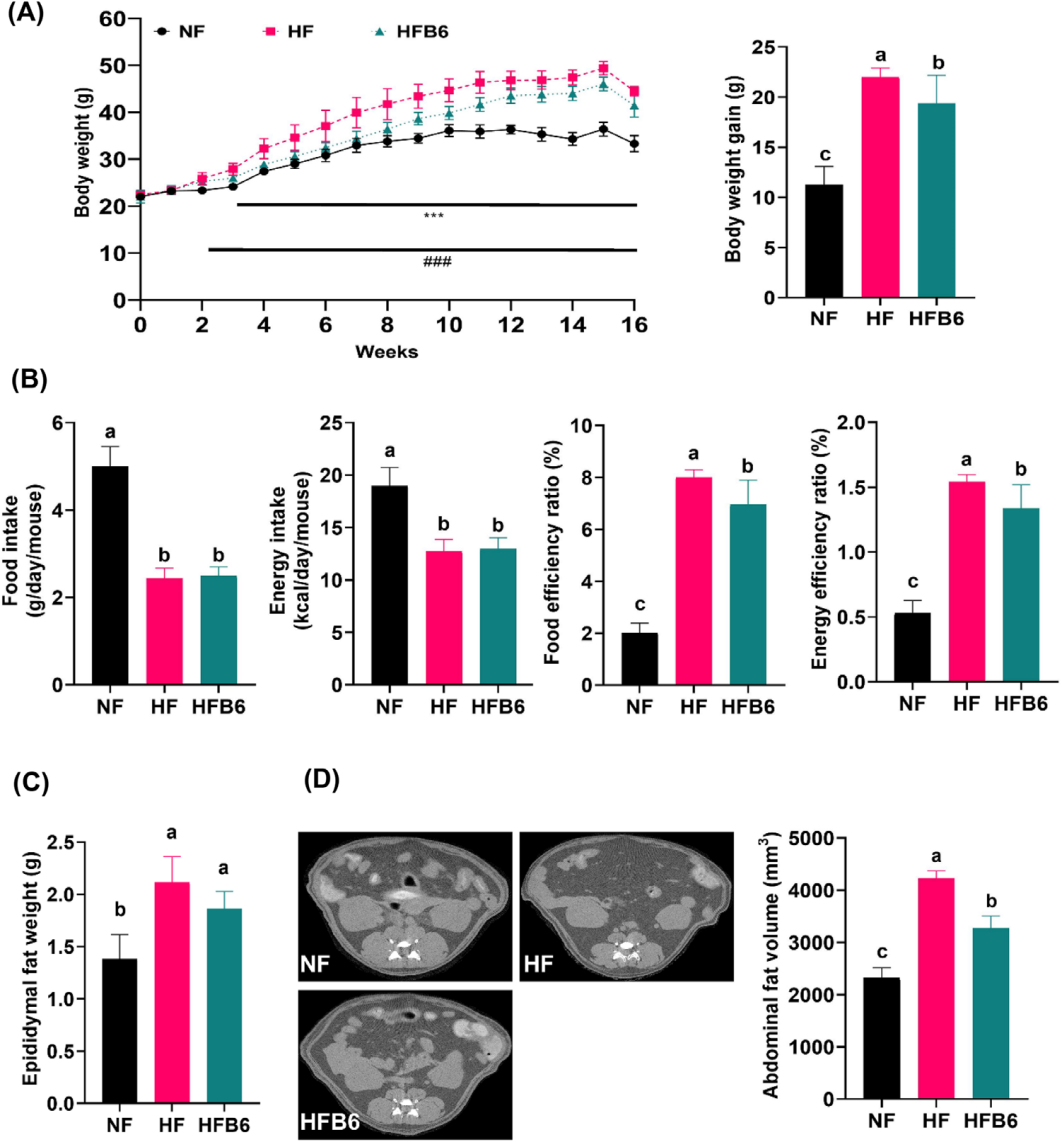
Effects of freeze‐dried whole blackcurrant on body weight, dietary intakes, and fat mass‐related parameters in high‐fat diet‐induced obese mice. (A) Changes in body weight during the experimental periods and final body weight gain. ^###^ *p* < 0.001 vs. NF; ^***^p < 0.001 versus HF. (B) Parameters related to dietary intakes. (C) Weight of epididymal fat. (D) Representative micro‐CT images and quantified volume of abdominal fat measured by micro‐CT. Dark‐gray, adipose tissue; Light‐gray, organs; White, bone. Data were expressed as mean ± SD (*n* = 8 per group). Significant differences assessed by one‐way ANOVA and Tukey's post hoc tests were indicated by different superscripts (a > b > c) above the error bars (*p* < 0.05). NF, Normal diet group; HF, High‐fat diet group; HFB6, High‐fat diet group with 6% freeze‐dried whole blackcurrant powder.

Changes in body weight and abdominal fat were observed to determine whether dietary intake of freeze‐dried whole BC could prevent obesity induced by an HFD. The HF group showed a significant increase in body weight from the second week to the 16th week of the experiment compared to the NF group (Figure 1A). Compared to the HF group, the HFB6 group began to show a significantly lower body weight from the third week, with approximately 12% reduction in HFD‐induced body weight gain. Food and energy intake did not differ significantly between the HF and HFB6 groups, whereas FER and EER were lower in the HFB6 group (Figure 1B).

Epididymal fat weight was lower in the HFB6 group than in the HF group, but the difference was not statistically significant (Figure 1C). In contrast, total abdominal fat volume was significantly reduced in the HFB6 group (Figure 1D). These results suggest that freeze‐dried whole BC effectively attenuates abdominal fat accumulation caused by an HFD without impacting epididymal fat weight, food intake, and energy intake.

### 3.2 Freeze‐Dried Whole BC Improves HFD‐Induced Lipid Profile Changes in the Liver and Serum

**FIGURE 2:**
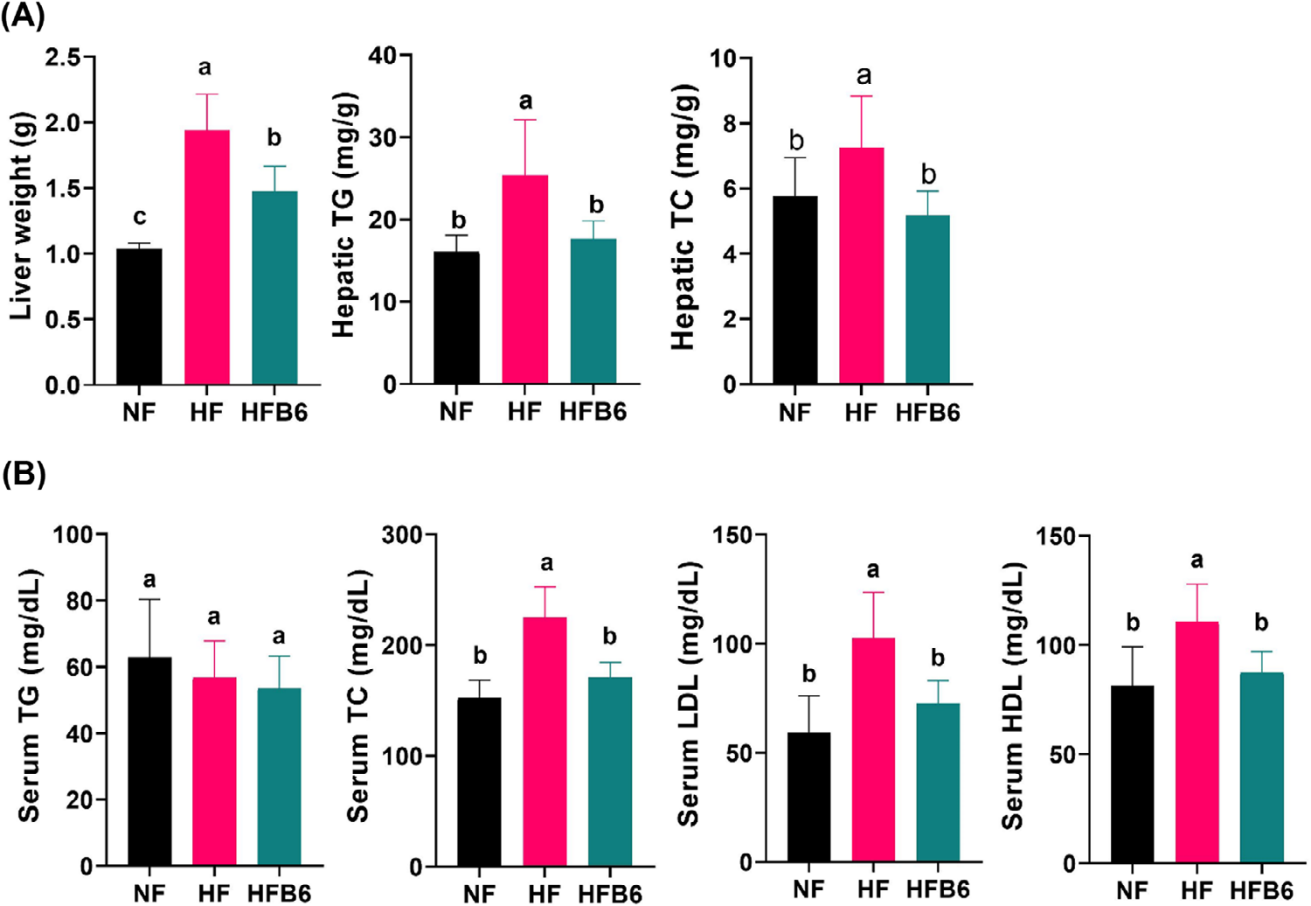
Effects of freeze‐dried whole blackcurrant on liver and serum lipid profiles in high‐fat diet‐induced obese mice. (A) Changes in weight and lipid profile (TG, TC) of the liver tissue. (B) Serum lipid profiles (TG, TC, LDL, HDL). Data were expressed as mean ± SD (*n* = 8 per group). Significant differences assessed by one‐way ANOVA and Tukey's post hoc tests were indicated by different superscripts (a > b > c) above the error bars (*p* < 0.05). NF, Normal diet group; HF, High‐fat diet group; HFB6, High‐fat diet group with 6% freeze‐dried whole blackcurrant powder.

The effect of freeze‐dried whole BC on HFD‐induced lipid levels in the liver and serum was examined (Figures 2A, B). The HF group showed a significant increase in liver tissue weight and levels of TG and TC in the liver compared with the NF group (Figure 2A). In addition, the serum TC, LDL, and HDL levels were higher in the HFD group than in the NF group (Figure 2B). Notably, the HFB6 group showed reduced liver tissue weight, hepatic TG and TC levels, and serum TC, LDL, and HDL levels, with these levels being similar to those in the NF group. These results confirm that an HFD causes abnormal lipid metabolism in the liver and serum, whereas dietary intake of freeze‐dried whole BC improves lipid metabolism.

### 3.3 Freeze‐Dried Whole BC Alleviates Damage to Colon Tissues Induced by an HFD

**FIGURE 3:**
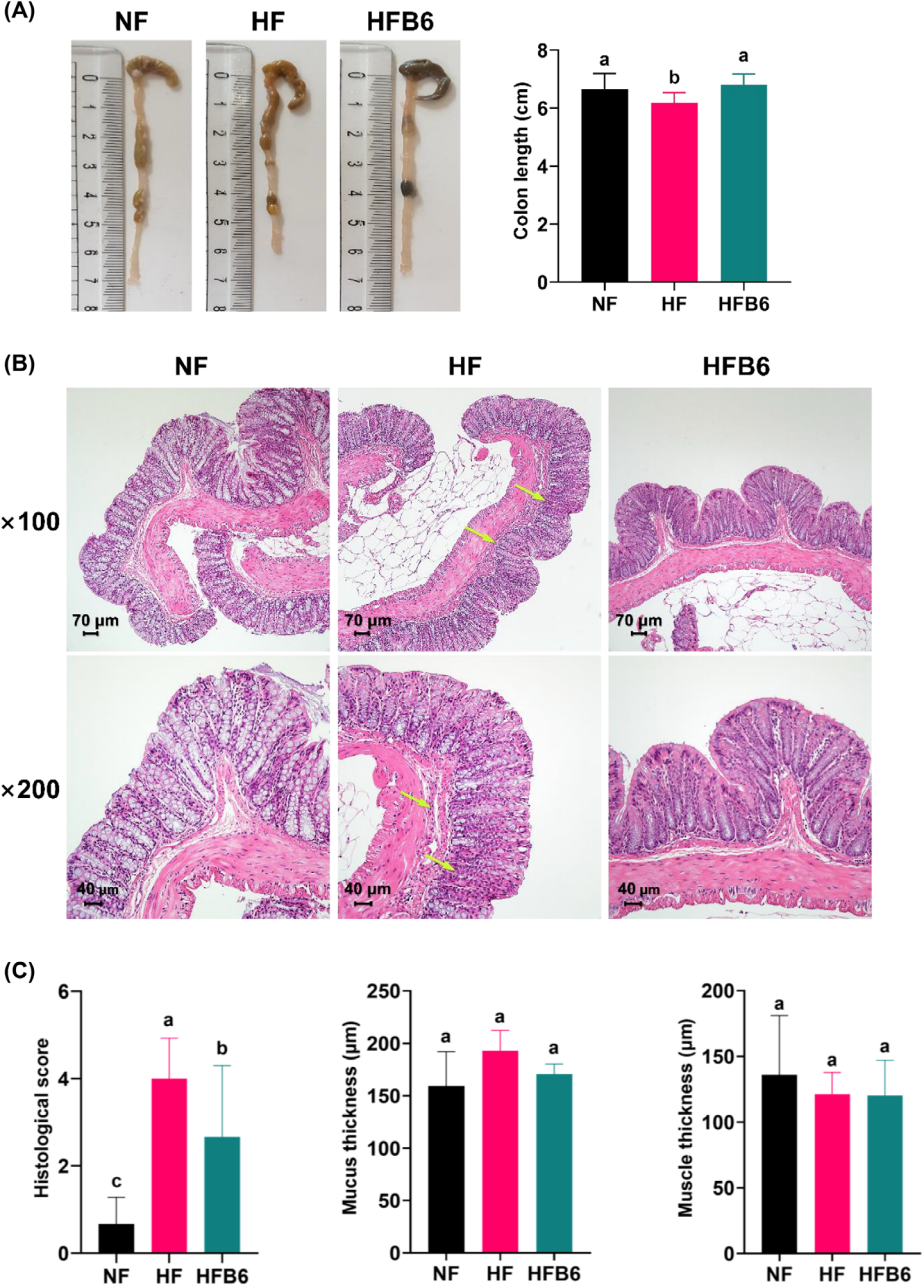
Effects of freeze‐dried whole blackcurrant on the damage of colonic tissue in high‐fat diet‐induced obese mice. (A) Representative photographs showing the length of the colon and its value. Data were expressed as mean ± SD (*n* = 8 per group). (B) Representative images of H&E‐stained colon sections (×100 and ×200; scale bars, 70 µm and 40 µm, respectively). (C) Histopathological parameters of colon sections. Yellow arrow, infiltration of inflammatory cell in mucosa and submucosal tissue. The results were expressed as the mean ± SD by evaluating the histological score in 5 fields of the stained slides of the colon in 4 animals per group. Significant differences assessed by one‐way ANOVA and Tukey's post hoc tests were indicated by different superscripts (a > b > c) above the error bars (*p* < 0.05). NF, Normal diet group; HF, High‐fat diet group; HFB6, High‐fat diet group with 6% freeze‐dried whole blackcurrant powder.

High‐fat diet (HFDs) have been reported to accelerate obesity by aggravating inflammation in the colon [3]. Therefore, morphological (Figure 3A) and pathological (Figures 3B, C) observations of colonic tissue were performed to confirm that dietary intake of freeze‐dried whole BC alleviates colonic tissue injury. The findings revealed that the colon length was significantly shorter in the HF group (6.71 ± 0.49 cm) by 7.8% compared to the NF group. In contrast, the HFB6 group exhibited a colon length of 6.81 ± 0.36 cm, similar to that of the NF group (Figure 3A).

Hematoxylin and eosin (H&E) staining of the colon in the NF group showed a well‐arranged crypt structure with goblet cells and normal mucosal and submucosal structures without inflammatory cell infiltration (Figures 3B, C). However, the HF group showed extensive colonic tissue damage, with inflammatory infiltration in the mucosal and submucosal tissues and loss of goblet cells. The HFB6 group mitigated alleviated goblet cell loss and inflammatory cell infiltration in the mucosa and submucosa with significantly reduced histological scores compared to the HF group. However, no significant differences were observed in mucus or muscle thickness among the groups.

### 3.4 Freeze‐dried Whole BC Suppresses the Overproduction of Inflammatory Factors in Colon Tissue and Serum in HFD‐induced Obesity

Pro‐inflammatory cytokine levels in colon tissue and serum were measured in HFD‐induced obese mice following dietary intake of freeze‐dried whole BC (Table 1). IL‐1β levels in colon tissues were significantly higher in the HF group than in the NF group. However, the HFB6 group showed significantly reduced levels compared to those of the HF group. No significant differences were noted in TNF‐α and IL‐6 in colon tissue among all groups.

**TABLE 1:** Effects of dietary intake of freeze‐dried whole blackcurrant on levels of pro‐inflammatory cytokines in colon and serum.

Ingredients (g)	Normal diet	High‐fat diet	
	NF	HF	HFB6
**Colon**			
TNF‐α (pg/µg protein)	1.40 ± 0.30^a^	1.43 ± 0.23^a^	1.29 ± 0.09^a^
IL‐1β (pg/µg protein)	1.08 ± 0.09^c^	1.65 ± 0.20^a^	1.37 ± 0.16^b^
IL‐6 (pg/µg protein)	1.71 ± 0.23^a^	1.56 ± 0.20 ^a^	1.58 ± 0.12^a^
**Serum**			
TNF‐α (pg/mL)	2.30 ± 1.81^c^	12.69 ± 2.73^a^	7.33 ± 2.81^b^
IL‐1β (pg/mL)	0.79 ± 0.29^b^	1.17 ± 0.29^a^	0.81 ± 0.15^b^
IL‐6 (pg/mL)	6.48 ± 1.67^b^	9.38 ± 1.78^a^	7.20 ± 1.35 ^ab^
Endotoxin (EU/mL)	7.15 ± 0.31 ^b^	7.75 ± 0.59^a^	7.00 ± 0.27^b^

Data were expressed as mean ± SD (*n* = 8 per group). Values with different superscripts within the same row are significantly different (*p* < 0.05) by one‐way ANOVA followed by Tukey's post hoc test. NF, Normal diet group; HF, High‐fat diet group; HFB6, High‐fat diet group with 6% freeze‐dried whole blackcurrant powder.

Serum levels of pro‐inflammatory cytokines (TNF‐α, and IL‐1β) and LPS were significantly higher in the HF group than in the NF group, whereas they were significantly lower in the HFB6 group than in the HF group. Thus, whole BC inhibited the levels of pro‐inflammatory cytokines and endotoxins that were excessively elevated by an HFD intake. Therefore, freeze‐dried whole BC improved the inflammatory response in the colon induced by an HFD, thereby reducing systemic inflammatory factors.

### 3.5 Freeze‐Dried Whole BC Inhibits Intestinal Mucosal Barrier Damage and Inflammatory Response in the Colon of HFD‐Induced Obese Mice

**FIGURE 4:**
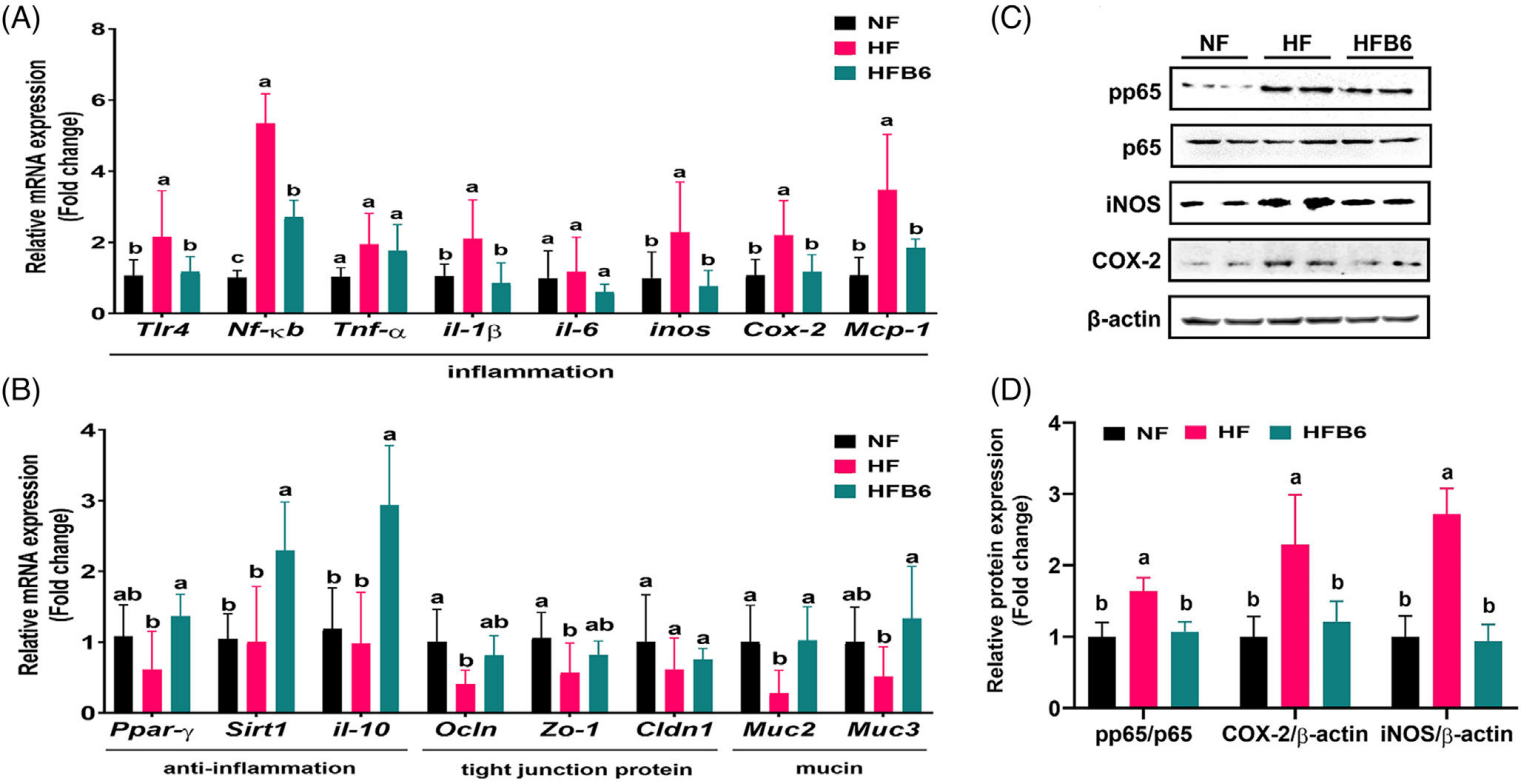
Effects of freeze‐dried whole blackcurrant on the inflammation‐related factors and intestinal mucosal barrier of the colon tissue in high‐fat diet‐induced obese mice. (A) Expression of genes of inflammation‐related factors in the colon. (B) Expression of anti‐inflammatory factors, tight junction protein and mucin‐related gene in the colon. (C, D) Expression levels of proteins related to the NF‐κB pathway associated with inflammation in the colon. Data were expressed as mean ± SD (*n* = 8 per group). Significant differences assessed by one‐way ANOVA and Tukey's post hoc tests were indicated by different superscripts (a > b > c) above the error bars (*p* < 0.05). NF, Normal diet group; HF, High‐fat diet group; HFB6, High‐fat diet group with 6% freeze‐dried whole blackcurrant powder.

High‐fat diet (HFDs) induce intestinal inflammation by adversely affecting the intestinal barrier system, including mucosal barriers such as tight junction proteins (TJ) and mucin (MUC), as well as the immune system, and gut microbiota [29]. Therefore, the effect of freeze‐dried whole BC on gene expression associated with intestinal barrier function in colon tissue was assessed using real‐time PCR (Figures 4A,B). Compared to the NF group, the HF group exhibited significantly higher mRNA expression levels of NF‐κB‐dependent inflammatory factors in colon tissue, including *Tlr4*, *Nf‐κb*, *il‐1β*, inducible nitric oxide synthase (*inos*), cyclooxygenase‐2 (*Cox‐2*), and monocyte chemoattractant protein‐1 (*Mcp‐1*) (Figure 4A). Additionally, the expression levels of TJ‐related genes, such as Occludin (*Ocln*) and zonula occludens‐1 (*Zo‐1*), as well as mucins (*Muc2*) were significantly decreased in the HF group (Figure 4B). Furthermore, the expression of anti‐inflammatory factor‐related genes in the HF group was not significantly different from that of the NF group but showed a tendency toward reduced peroxisome proliferator‐activated receptor (*Ppar*)‐γ levels (Figure 4B).

In contrast, although the HFB6 group was fed an HFD, the expression levels of NF‐κB‐dependent inflammatory factors (upregulated in the HF group) and TJ and mucin genes (downregulated in the HF group) were restored to levels comparable to those of the NF group. In addition, the expression levels of genes associated with anti‐inflammatory factors were higher in the HFB6 group than in the HF group.

To examine the mechanism underlying inflammation reduction in the colon, the expression of key proteins in the NF‐κB pathway was analyzed using western blotting (Figures 4C, D). Compared to the NF group, the HF group exhibited significantly increased phosphorylation of NF‐κB p65 (pp65) in colon tissue. In addition, the protein expression levels of COX‐2 and iNOS, downstream enzymes of NF‐κB, were markedly elevated in the HF group. In contrast, in the HFB6 group, the overactivation of pp65 induced by HFD was suppressed, leading to reduced COX‐2 and iNOS expression. These findings revealed that freeze‐dried whole BC lowers inflammatory responses by suppressing the NF‐κB signaling pathway in the colon.

Moreover, chronic inflammation in the colon is known to contribute to fibrosis [30]. Further analysis of fibrosis‐related genes in the colon revealed that the HF group exhibited higher collagen deposition and elevated mRNA expression of collagen type 1 (*Col1a1*) and connective tissue growth factor (*Ctgf*) compared to the NF group (Figure S1). In contrast, the HFB6 group showed a significant reduction in these levels.

### 3.6 Freeze‐Dried Whole BC Alters Gut Microbiota Diversity in HFD‐Induced Obese Mice

**FIGURE 5:**
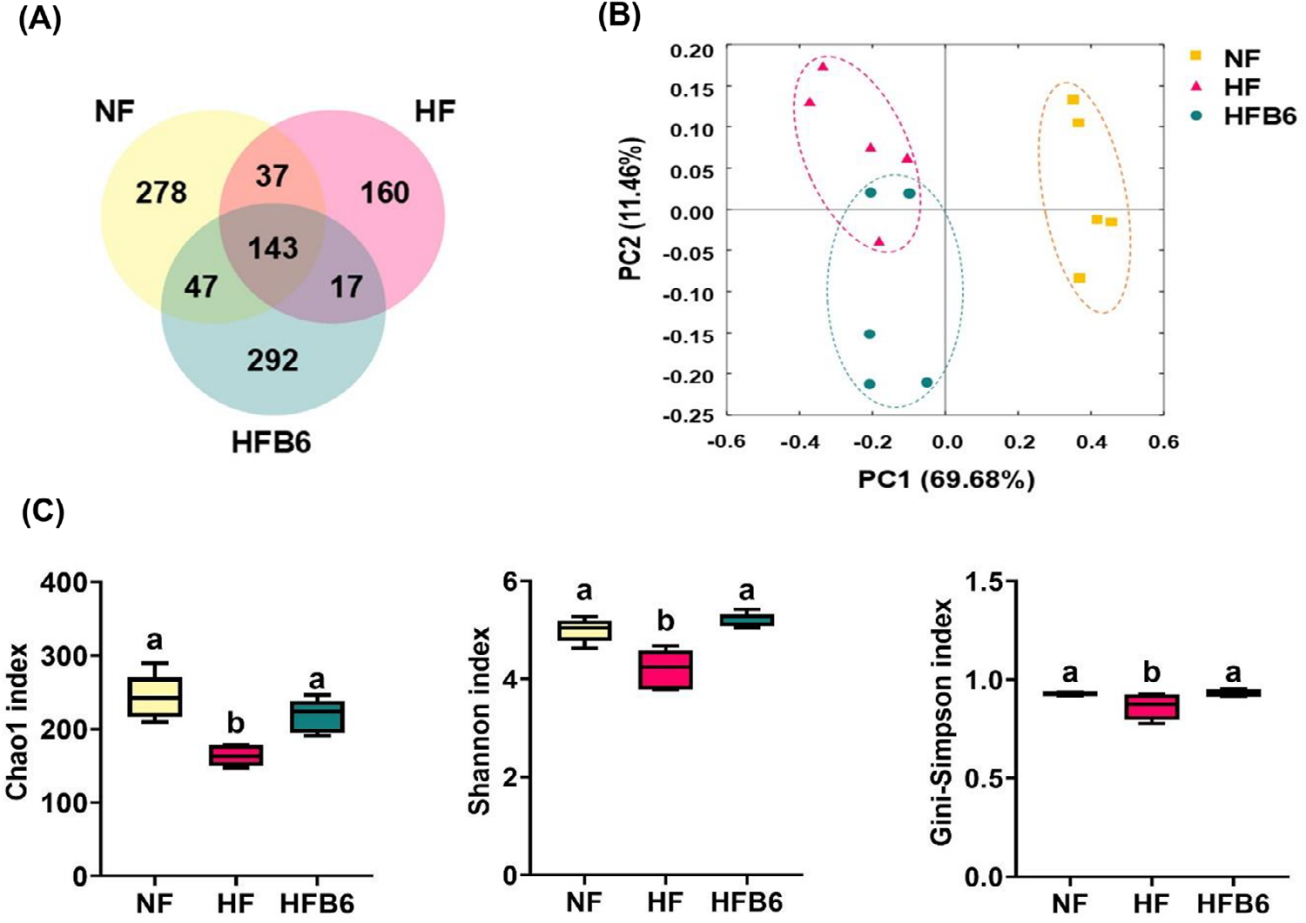
Effects of freeze‐dried whole blackcurrant on the diversity of gut microbiota in high‐fat diet‐induced obese mice. (A) Venn diagram for amplicon sequence variant (ASV). (B) Beta‐diversity using principal coordinate analysis (PCoA) based on weighted UniFrac. (C) Alpha‐diversity values, such as Chao1, Shannon index, and Gini‐Simpson index. Data are shown as box‐and‐whisker plots, where the center line in the box denotes the median, the box represents the interquartile range (25th–75th percentiles), and the whiskers indicate the minimum and maximum values (*n* = 5 per group). Significant differences assessed by one‐way ANOVA and Tukey's post hoc tests were indicated by different superscripts (a > b > c) above the error bars (*p* < 0.05). NF, Normal diet group; HF, High‐fat diet group; HFB6, High‐fat diet group with 6% freeze‐dried whole blackcurrant powder.

Next, we examined whether freeze‐dried whole BC influenced gut microbiota diversity in mice with HFD‐induced obesity (Figure 5). A Venn diagram was generated to show the number of shared and individual ASVs (Figure 5A). The number of ASVs, representing the number of species, shared by the three groups was 143. The total ASVs in the NF, HF, and HFB6 groups were 505, 357, and 499, respectively, and the HFB6 group had more abundant total ASVs than the HF group.

Beta‐diversity, which represents the phylogenetic relationships of the different bacterial taxa among the experimental groups was supported via PCoA based on the weighted UniFrac distance metric (Figure 5B). Substantial variations existed between the NF and HFD (including HF and HFB6) groups along the principal component (PC)1 dimension (69.68%), indicating that the HFD was the primary factor affecting microbiota composition. In addition, based on the PC2 dimension (11.46%), the HFB6 group showed a cluster pattern distinct from that of the HF group.

Alpha‐diversity, which represents the evenness and richness of the gut microbiota was assessed using the Chao1, Shannon, and Gini–Simpson indices (Figure 5C). The HF group showed a significant decrease in all alpha‐diversity values compared to the other groups. These results revealed that BC effectively restored the evenness and richness of the gut microbiota altered by an HFD.

### 3.7 Freeze‐Dried Whole BC Modulated Gut Microbiota Composition in HFD‐Induced Obese Mice

Changes in the taxonomic composition of the gut microbiota were analyzed to examine how the intake of freeze‐dried whole BC modulates dysbiosis of the gut microbiota triggered by an HFD (Figure 6). At the phylum level, *Bacteroidetes*, *Firmicutes*, and *Proteobacteria* were the dominant microbiota in all groups, although their relative abundances differed in each group (Figure 6A). In particular, the HFD groups showed significantly lower relative abundances of *Bacteroidetes* and higher relative abundances of *Firmicutes* than the NF group (Figure 6B). Notably, the ratio of *Firmicutes* to *Bacteroidetes* (F/B ratio) was significantly lower in the HFB6 group than in the HF group (Figure 6C).

FIGURE 6Effects of freeze‐dried whole blackcurrant on the relative abundance composition of gut microbiota changed by a high‐fat diet. (A) Histogram of relative abundance of gut microbiota at the phylum level. (B) Bar graph of relative abundance of major phylum. (C) Box‐and‐whisker plot of F/B ratio. The center line in the box denotes the median, the box represents the interquartile range (25th‐75th percentiles), and the whiskers indicate the minimum and maximum values (*n* = 5 per group). (D) Histogram of relative abundance of gut microbiota at the genus level. (E, F) LEfSe analysis (LDA score > 4, *p* < 0.05) and cladogram to identify major differentially abundant microbiota between groups at the taxonomic level from phylum to genus. Different colored nodes represent characteristically altered bacteria in each group. Yellow nodes represent bacteria with no significant differences. From the inside to the outside, it represents the phylum, class, order, family, and genus. (G, H) Histogram and heatmap for comparison of relative abundance (> 2%) of gut microbiota between groups at the species level. Heatmap was shown as normalized relative abundance. Data were expressed as mean ± SD (*n* = 5 per group). Significant differences assessed by one‐way ANOVA and Tukey's post hoc tests were indicated by different superscripts (a > b > c) above the error bars (*p* < 0.05). NF, Normal diet group; HF, High‐fat diet group; HFB6, High‐fat diet group with 6% freeze‐dried whole blackcurrant powder.

**Figure:**
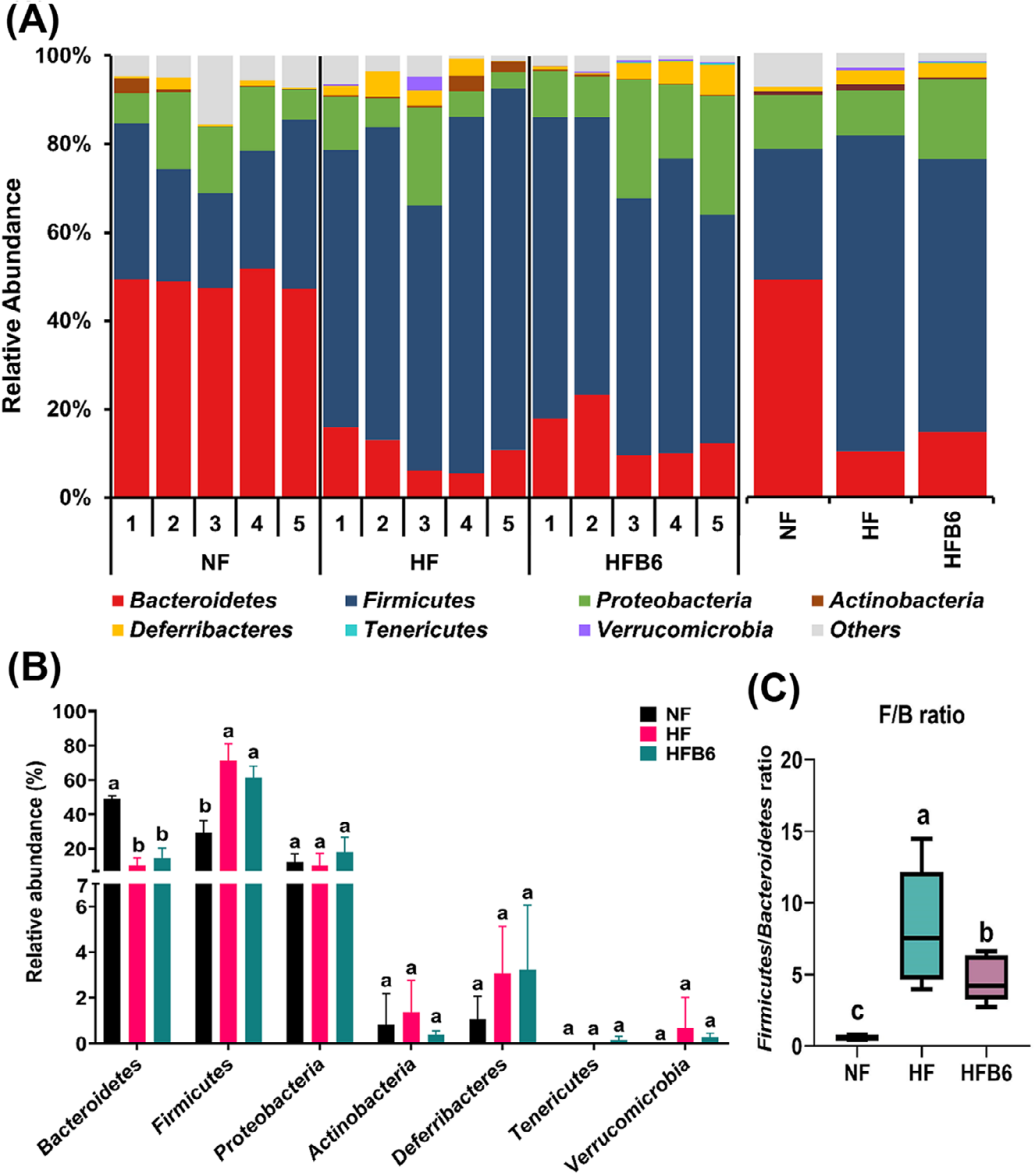

**Figure:**
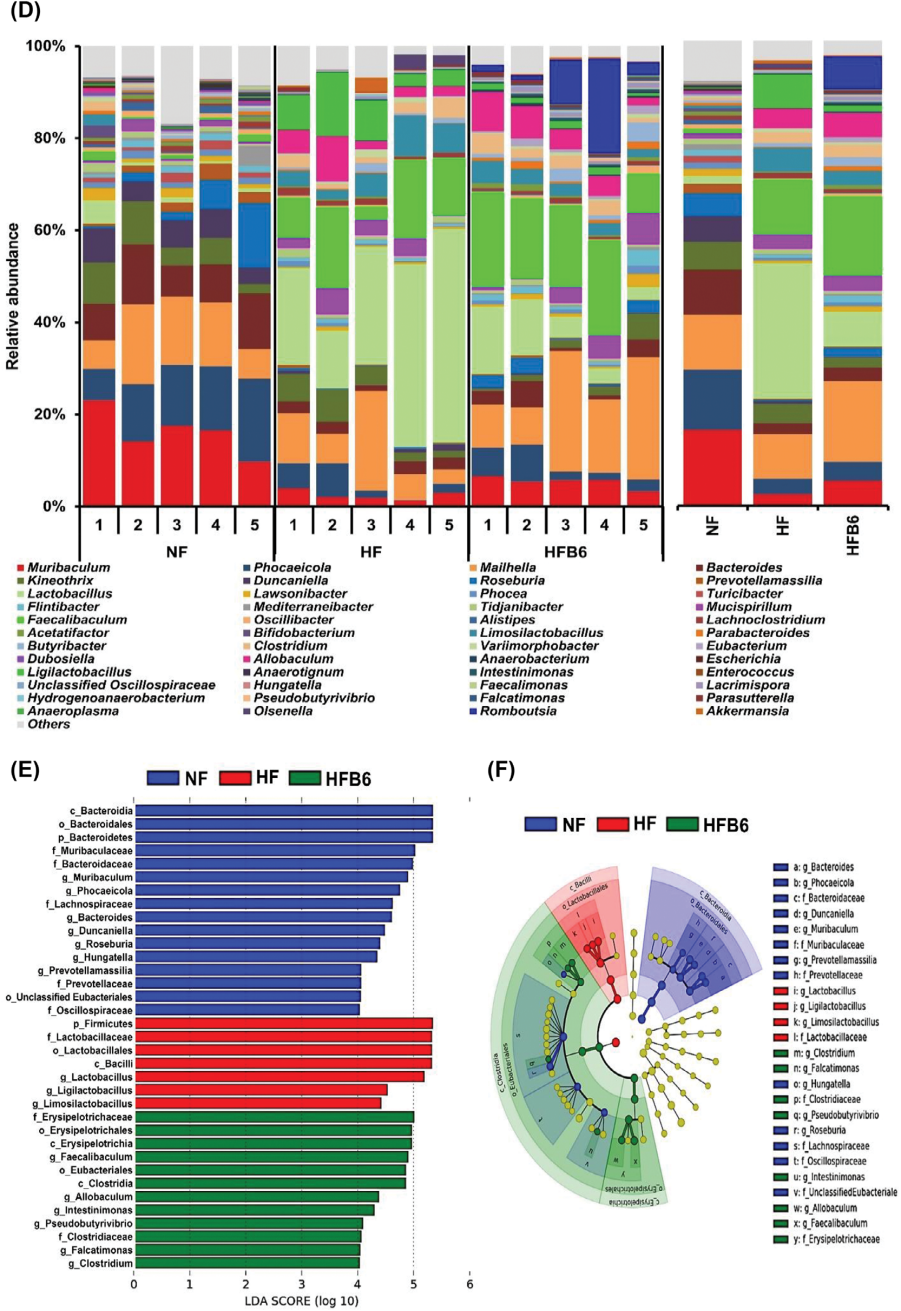

**Figure:**
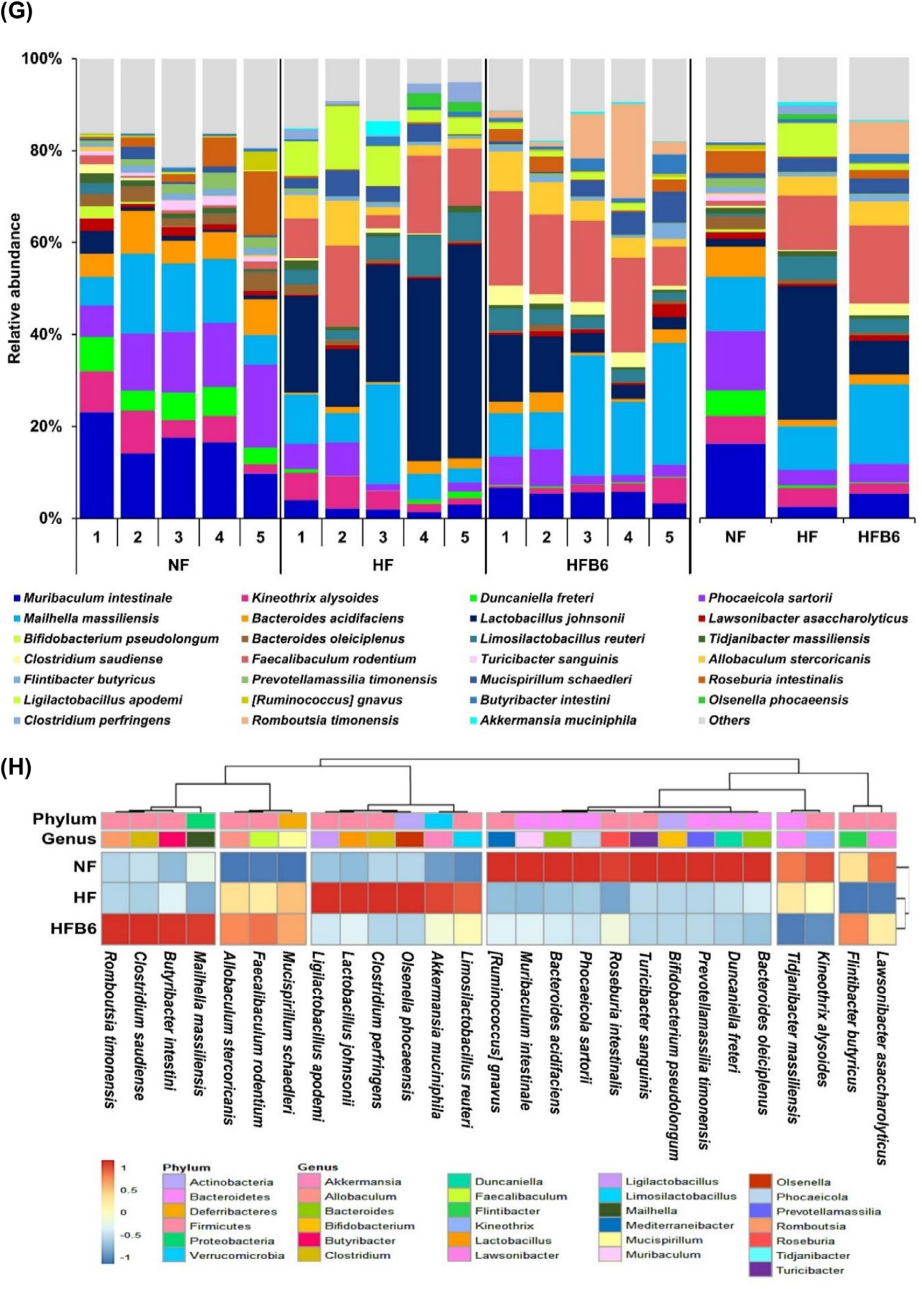

At the genus level, the dominant bacteria in the NF group were *Muribaculum*, *Phocaeicola*, *Maihella*, and *Bacteroides* (Figure 6D). In contrast, *Lactobacillus*, *Faecalibaculum*, and *Mailhella* were the three most abundant bacteria in the HF and HFB6 groups. However, the HFB6 group showed a lower relative abundance of *Lactobacillus* and *Faecalibaculum* and higher relative abundance of *Maihella* than the HF group. Furthermore, LEfSe analysis was performed to distinguish the prominent taxa of the gut microbiota among the three groups (Figures 6E, F). The HF group showed the highest differential abundance of the genera *Lactobacillus*, *Ligilactobacillus*, and *Limosilactobacillus* belonging to the phylum *Firmicutes*. The HFB6 group had multiple differential communities that gathered in the familiae *Erysipelotrichaceae* (genera *Faecalibaculum*, *Allobaculum*), *Eubacteriales incertae sedi* (genus *Intestinimonas*), *Lachnospiraceae* (genera *Pseudobutyrivibrio*, *Falcatimonas*), and *Clostridiaceae* (genus *Clostridium*), which correspond to the phylum *Firmicutes*.

In addition, bacteria that changed significantly at the species level were examined to assess gut microbiota with a relative abundance of ≥ 2% (Figures 6G, H). In the HF group, the species with a particular increase compared to other groups were *Limosilactobacillus reuteri* and *Ligilactobacillus apodeme*. In addition, *Lawsonibacter asaccharolyticus* and *Flintibacter butyricus* were lower in the HF group, but higher in the NF and HFB6 groups. Specifically, *Butyribacter intestini* was considerably elevated in the HFB6 group. These findings revealed that dysbiosis of the gut microbiota caused by an HFD could be partially improved by incorporating freeze‐dried whole BC into the diet.

### 3.8 The Relative Abundance of Gut Microbiota was Correlated with Obesity and Colonic Inflammation‐Related Parameters in Mice on an HFD

**FIGURE 7:**
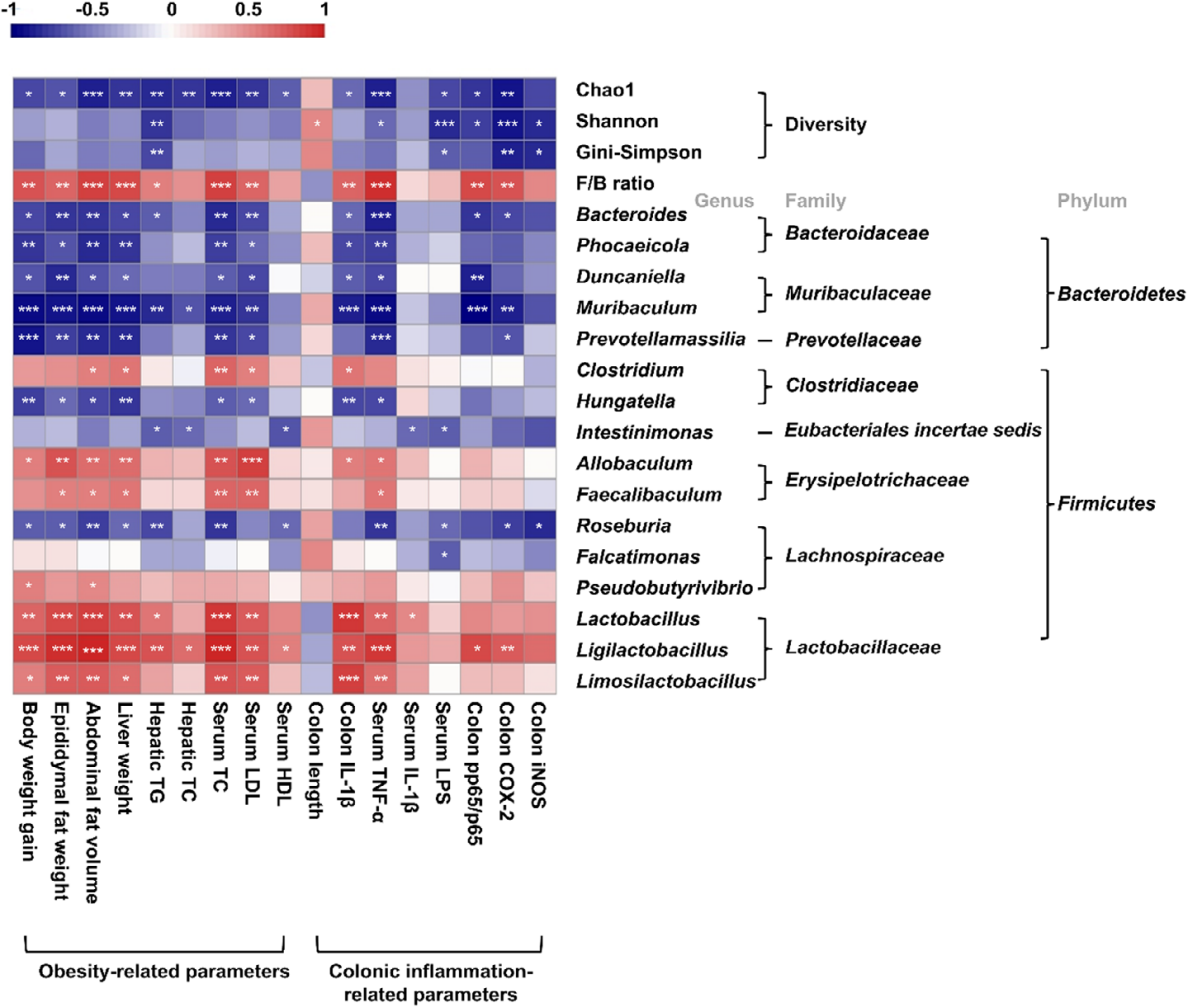
Spearman's correlation heatmap between gut microbiota and biomarkers related to obesity and colonic inflammation in HFD‐induced obese mice. Red squares represent positive correlations, and blue squares represent negative correlations. The darker the color, the stronger the correlation, and statistical significance is shown as follows: * *p* < 0.05, ** *p* < 0.01, *** *p* < 0.001.

Spearman correlation analysis was used to estimate the relationship between the gut microbiota and biomarkers associated with obesity and colonic inflammation (Figure 7). The gut microbiota used in the correlation analysis were selected only for genera that showed significant differences among the groups, based on the LEfSe analysis. The gut microbial diversity indexes were further analyzed to determine its correlation with clinical parameters.

The alpha‐diversity index, especially Chao1 was negatively correlated with almost all clinical parameters except for colon length. In contrast, the F/B ratio showed a positive correlation with obesity‐related indicators such as body weight gain, fat accumulation, hepatic TG, and serum lipids, and colon IL‐1β, serum TNF‐α, and colon NF‐κB pathway‐related protein expressions. The relative abundance of all genera belonging to the phylum *Bacteroidetes* and the genera *Hungatella*, *intestinimonas*, and *Roseburia*, which correspond to the phylum *Firmicutes*, were negatively correlated with biomarkers related to obesity and colonic inflammation. Conversely, the relative abundance of *Lactobacillus*, *Ligilactobacillus*, and *Limosilactobacillus* exhibited a strong positive correlation with most variables associated with obesity and cytokines in the colon and serum. In summary, parameters associated with obesity and colon inflammation were correlated with changes in gut microbial diversity and structure.

## 4 Discussion

Dietary plants are an ongoing strategy to combat increasing obesity [2]. Previous studies have documented that the whole berries, as opposed to their single constituents, exert positive and synergistic effects on the immune system [19, 31]. The present study investigated the impact of freeze‐dried whole BC on obesity‐related factors in mice on HFD, and confirmed the amelioration of obesity and associated injury to the colonic mucosal barrier function by regulating the HFD‐induced gut microbiota changes and colonic inflammation, leading to relief in a chronic systemic low‐grade inflammatory state and obesity‐related metabolic disorders.

An HFD containing 6% freeze‐dried whole BC prevented obesity and lipid profile disturbances in the serum and liver without changes in food and energy intake. Previous studies have corroborated the protective effects of 6% freeze‐dried whole BC powder and ACNs extracted from BC on HFD‐induced obesity, NASH, and hyperlipidemia [17, 20]. In the current study, one mouse in the HFB6 group consumed approximately 2.5 g food/d, which is equivalent to approximately 3.5 mg of ACN in BC [24]. In a previous study, oral administration of ACNs extracted from BC at a dose of 150 mg/kg body weight in mice fed a 45% HFD improved body weight and lipid metabolism [17]. This is equivalent to a mouse with a body weight of 35 g consuming 5.25 mg/d of ACN extracted from BC. As this dose is higher than that used in the present study, ACN consumption at a lower dose in the whole form may be more beneficial against obesity than in a compound form. Another study reported crude ACN extracts from blueberries to exert more prominent antitumor effects than the purified extracts; this was attributed to the synergistic effects of proteins, polysaccharides, and other phytochemicals [19, 32]. Similarly, the antiobesity effects of whole BC are believed to result from interaction of various compounds, such as proanthocyanins, vitamin C, and ACN [13, 24].

The intestine maintains a barrier system that protects the host from the external environment and consists of microbial, mucus, epithelial, and immunological barriers [29]. The colonic mucus layer mainly comprises MUC2, a gel‐forming secretory mucin secreted by goblet cells, and MUC3, a membrane‐bound mucin [33].

In the intestinal epithelial layer, various cell types, including intestinal epithelial cells, form TJ complexes composed of claudin‐1 (CLDN1), ZO‐1, and OCLN, thereby establishing a physical barrier that regulates intestinal permeability [29]. However, dietary fat is known to directly cause barrier dysfunction by reducing the contents of TJs and altering the properties of the intestinal mucus [3, 6, 29]. Defects in the colonic mucus layer were also observed in genetically obese *ob*/*ob* mice when the diet was closely controlled [34]. In another study, when obese mice were fed a complex of delphinidin and cyanidin for 4 weeks, the expression of TJ proteins increased in the colon, resulting in the improvement of mucosal barrier function [35]. BC contains delphinidin and cyanidin glycosides [13]. Freeze‐dried whole BC showed a mitigation of histopathological and morphological damage and an increase in the mRNA expressions of mucosal barrier‐related components in the colon of HFD‐fed mice, which may involve ACN in BC.

In addition, the destruction of the intestinal mucus and epithelial layers increases intestinal permeability, facilitating the influx of endotoxins such as LPS derived from gut microbiota into the blood. This may induce metabolic inflammation mediated by TLR signaling, ultimately leading to the development of metabolic disorders including obesity [3, 29, 36]. Furthermore, obesity is characterized by the secretion of inflammatory cytokines (such as TNF‐α, IL‐1β, and IL‐6), which promotes macrophage infiltration into the liver and adipose tissues, contributing to systemic inflammation [4, 37]. In the present study, freeze‐dried whole BC supplementation reduced circulating TNF‐α, IL‐1β, and LPS levels in HFD‐fed mice. This indicates that freeze‐dried whole BC attenuates metabolic inflammation by contributing to the maintenance of barrier function.

Intestinal inflammation is characterized by the secretion of inflammatory cytokines, recruitment of immune cells, and expression of various inflammatory mediators [9, 38, 39, 40]. Recovery from intestinal inflammation ameliorates metabolic disorders by reducing inflammation in visceral adipose tissue [9]. The NF‐κB signaling pathway plays a key role in regulating inflammatory responses in colon, which is activated by upstream signals such as TLR‐4, a pattern recognition receptor that is directly stimulated by endotoxins such as SFAs and LPS [3, 41, 42]. NF‐κB signaling pathway activation upregulates the gene and protein expression of pro‐inflammatory cytokines (TNF‐α and IL‐1β), enzymes (iNOS and COX‐2), and chemokines (MCP‐1), resulting in an excessive inflammatory response [3, 29, 41]. However, PPAR‐γ and Sirt1 can reduce excessive inflammatory responses by inhibiting the acetylation and phosphorylation of NF‐κB p65 [43]. *Tlr‐4, Nf‐κb, Cox‐2, inos, il‐1β*, and *Mcp‐1* expressions were downregulated and *il‐10, Ppar‐γ*, and *Sirt1* expressions were upregulated in the colon in the HFB6 group compared to that in the HF group. These changes suppressed pp65 and reduced the production of the pro‐inflammatory enzymes iNOS and COX‐2, as well as the pro‐inflammatory cytokine IL‐1β, which contributes to increasing TJ permeability of the intestinal epithelial layer by activating NF‐κB in the colon [39]. Furthermore, ACNs extracted from *Lycium ruthenicum* in mice ameliorated intestinal inflammation and improved barrier function by inhibiting the activation of the NF‐κB signaling pathway in the colon [12]. Collectively, these results indicate that freeze‐dried whole BC, which contains ACNs, improves barrier function by reducing colonic inflammation through the suppression of the excessive activation of the NF‐κB pathway induced by an HFD.

Notably, chronic colonic inflammation leading to intestinal fibrosis has been linked to long‐term consumption of an HFD [30]. This investigation revealed that freeze‐dried whole BC reduced collagen accumulation and downregulated *Ctgf* and *Col1a1* expression, both of which are associated with intestinal fibrosis. Therefore, further research is required to investigate the effects of freeze‐dried whole BC on fibrosis resulting from long‐term chronic colonic inflammation.

Gut microbiota dysbiosis is associated with various metabolic diseases, and low gut microbial diversity and increased F/B ratio have especially been reported in obesity and related diseases [3, 44]. This imbalance can be changed by dietary intake containing polyphenols (including ACN), thereby preventing metabolic diseases [12, 17, 27, 41]. In previous studies, a diet rich in *Lycium ruthenicum*, purple sweet potato, and ACN improved intestinal inflammation and obesity by mitigating gut microbiota imbalance through the restoration of the gut microbial diversity and a decrease in the increased F/B ratio [12, 27, 45]. This was consistent with the results of our study, indicating that ACN in the freeze‐dried whole BC may have partially contributed to the restoration of gut microbial dysbiosis.

Some human and animal studies have reported that the relative abundance of *Lactobacillus* belonging to the phylum *Firmicutes* is higher in the intestine of obese individuals [12, 36, 46]. Similarly, increases in *Lactobacillus*, *Ligilactobacillus*, and *Limosilactobacillus* were observed in the HF groups. However, the role of commensal *Lactobacillus* spp. in the colon is still not fully understood. For example, *Lactobacillus* spp. residing in the colon have been associated with both weight gain and weight loss, and these effects appear to be influenced by genetic differences in lipid and carbohydrate metabolism [47]. Furthermore, *L. reuteri* isolated from normal mice prevented obesity, whereas the same species isolated from obese mice did not, thus revealing that *L. reuteri* may have complex interactions depending on the host [48]. In addition, an HFD increased reactive oxygen species (ROS) levels in the colon, which induced colonization by *L. sakei* strains with high catalase activity. However, increased levels of these strains were not the cause of obesity [49].

Polyphenols have been reported to contribute to the improvement of redox balance by neutralizing reactive species in the body through improved antioxidant activity of catalase in obese adults [50]. Freeze‐dried whole BC reduced the elevated levels of *L. reuteri* caused by an HFD, suggesting that the polyphenols in BC likely restored redox homeostasis by indirectly neutralizing the ROS generated by an HFD. Further research is required to elucidate the mechanisms of action.

Another notable finding was that the genera *Faecalibaculum*, *Allobaculum*, *Intestinimonas*, and *Pseudobutyrivibrio* were identified as the differential gut microbiota In the HFB6 group. The relative abundances of *Faecalibaculum*, *Allobaculum*, and *Pseudobutyrivibrio* increased with the intake of polyphenol‐containing extracts from mulberry leaf, ginger, and pomegranate, respectively [51, 52, 53]. In addition, *Intestinimonas* produces butyrate and improves host metabolism, particularly in relation to obesity [54]. Polyphenols function as prebiotics, promoting the growth of gut microbes and enhancing the production of short‐chain fatty acids (SCFAs), including butyrate [55]. Butyrate, a primary energy source for colonic epithelial cells, maintains intestinal homeostasis by suppressing oxidative stress and NF‐κB/NLRP3 activation in the intestine and activating PPAR‐γ pathway, as well as preventing obesity and colitis by enhancing barrier function [56, 57, 58]. Although we did not measure butyrate concentrations, freeze‐dried whole BC consumption increased the abundance of the butyrate‐producing bacteria including *Butyribacter intestini*, *Lawsonibacter asaccharolyticus*, and *Flintibacter butyricus* [59, 60, 61], suggesting that metabolites from these strains may have contributed to the alleviation of obesity and colonic inflammation.

Previous research on BC has predominantly focused on ACN‐rich extracts, leaving a knowledge gap regarding the health benefits of consuming whole BC. This study may contribute to filling this research gap by evaluating the effects of consuming whole BC in an HFD‐induced obesity and demonstrating simultaneous improvements in obesity, colonic inflammation, and gut microbiota restructuring. These findings provide a foundational basis for future clinical applications of whole‐BC interventions.

Despite these findings, several limitations have been acknowledged. First, although whole BC intake increased microbial taxa associated with SCFA production, these metabolites were not directly quantified. Future research utilizing comprehensive metabolomic methodologies is necessary to elucidate the functional implications of these microbial alterations and their association with host metabolic health. Second, the observed increase in gut‐resident *L. reuteri* under HFD conditions may reflect an adaptive response to diet‐induced gut stress. This underscores the need for strain‐level functional studies to define the context‐dependent role of specific microbes in obesity‐related colonic inflammation. Finally, as gut microbiota composition has been reported to vary by age and sex, further investigations, including dose‐response trials and clinical studies, are necessary to validate the generalizability and therapeutic efficacy of whole BC‐based dietary strategies.

Nevertheless, our findings demonstrate that whole BC modulates gut microbiota diversity and mitigates excessive colonic inflammation, thereby reducing circulating endotoxin levels and alleviating obesity, a chronic low‐grade inflammatory state. These results suggest that whole BC may be a potentially effective dietary strategy for managing obesity and its associated metabolic complications.

## 5 Conclusion

In conclusion, dietary intake of whole BC improved obesity‐related parameters, including body weight gain, lipid accumulation in the liver and adipose tissue, and lipid profiles in the liver and serum. Additionally, whole BC restored mucosal barrier function by increasing the gene expression of TJ and mucin in the colon. It also inhibited the overactive NF‐κB signaling pathway induced by an HFD, reducing the production of pro‐inflammatory cytokines and enzymes in the colon, thereby contributing to the reduction of systemic inflammation. Moreover, whole BC restored gut microbiota diversity altered by an HFD and decreased the F/B ratio. Furthermore, it ameliorated gut microbiota dysbiosis by modifying the microbial composition associated with obesity. Therefore, whole BC demonstrated beneficial effects on colonic inflammation and obesity by regulating the gut microbiota, highlighting its potential as a preventative dietary supplement against obesity and colonic inflammation.

## Conflicts of Interest

The authors declare no conflict of interest.

## Supporting information




**Supporting File**: mnfr70462‐sup‐0001‐SuppMat.pdf.

## Data Availability

The data supporting the findings of this study are available within the article and its supporting information files and from the corresponding author upon reasonable request.
