# Supplementary material for: Whole Blackcurrant (Ribes nigrum) Alleviates High‐Fat Diet Induced‐Obesity and Colonic Inflammation by Modulating the Gut Microbiota in Mice
Source: Mol Nutr Food Res. 2026 Apr 15;70:e70462. doi: 10.1002/mnfr.70462 (PMC13081517; doi:10.1002/mnfr.70462)
Supplement: Supplementary file 1 — Supporting File: mnfr70462‐sup‐0001‐SuppMat.pdf. [file MNFR-70-e70462-s001.pdf]

Table S1. Composition of the diet used in the experiment

Ingredients (g)	Normal diet	High-fat diet	
	**NF**	**HF**	**HFB6**
Casein	200.0	200.0	197.0
L-Cystine	3.0	3.0	3.0
Corn starch	506.2	0.0	0.0
Maltodextrin	125.0	125.0	113.5
Sucrose	72.8	72.8	52.8
Cellulose	50.0	50.0	39.0
Soybean Oil	25.0	25.0	25.0
Lard	20.0	245.0	245.0
Mineral Mix	10.0	10.0	10.0
Vitamin Mix	10.0	10.0	10.0
Choline Bitartrate	2.0	2.0	2.0
Dicalcium phosphate	13.0	13.0	13.0
Calcium carbonate	5.5	5.5	5.5
Potassium citrate	16.5	16.5	16.5
Freeze-dried whole blackcurrant powder	-	-	46.5
Total	1059	777.8	778.8
**kcal/g**	3.8	5.2	5.2
**Energy (kcal %)**			
Carbohydrate	70	20	20
Protein	20	20	20
Fat	10	60	60

NF, Normal diet group; HF, High-fat diet group; HFB6, High-fat diet group with 6% freeze-dried whole blackcurrant powder.

Table S2. Primer list

Gene	Forward primer (5'-3')	Reverse primer (5'-3')
Tlr-4	GGC AGC AGG TGG AAT TGT AT	AGG CCC CAG AGT TTT GTT CT
Nf-κb	GAA TTC AGT CAC TGG CCT CC	TTC AAG ACA AAG GAG GTC TGT TT
Cox-2	ACC CCC TGC TGC CCG ACA CCT	CCA GCA ACC CGG CCA GCA ATC
inos	GCC TTG GCT CCA GCA TGT ACC CTC AG	CCT GCC CAC TGA GTT CGT CCC CTT C
Tnf-α	ACG GCA TGG ATC TCA AAG AC	GTG GGT GAG GAG CAC GTAGT
Il-1β	GAC CTT CCA GGA TGA GGA CA	AGC TCA TAT GGG TCC GAC AG
Il-6	AAC GAT GAT GCA CTT GCA GA	GAG CAT TGG AAA TTG GGG TA
Mcp-1	CAA GAG TGA ATC CAC ACA ACA G	GTA GGA GTC AAC TCA GCT TTC T
Il-10	GGT TGC CAA GCC TTA TCG GA	ACC TGC TCC ACT GCC TTG CT
Ppar-γ	GCC CAC CAA CTT CGG AAT C	TGC GAG TGG TCT TCC ATC AC
Sirt1	AAA GGA ATT GGT TCA TTT ATC AGA G	TTG TGG TTT TTC TTC CAC ACA
Occludin	ATG TCC GGC CGA TGC TCT C	TTT GGC TGC TCT TGG GTC TGT AT
Zo-1	ACC CGA AAC TGA TGC TGT GGA TAG	AAA TGG CCG GGC AGA ACT TGT GTA
Claudin-1	TCT ACG AGG GAC TGT GGA TG	TCA GAT TCA GCA AGG AGT CG
Muc-2	GCT GCT CAT TGA GAA GAA CGA TGC	CTC TCC AGG TAC ACC ATG TTA CCA GG
Muc-3	CCA CCA CTG TTG AAG TCA CAA	CAG AAC CCT CCG TTC ATA CAA
αsma	CTG ACA GAG GCA CCA CTG AA	CAT CTC CAG AGT CCA GCA CA
Ctgf	CAA AGC AGC TGC AAA TAC CA	GGC CAA ATG TGT CTT CCA GT
Col1a1	AAG AGG CGA GAG AGG TTT CC	AGA ACC ATC AGC ACC TTT GG
Gapdh	GGT AAG GTC GGT GTG AAC G	CTC GCT CCT GGA AGA TGG TG

Tlr-4, toll-like receptor 4; *Nf-κb*, nuclear factor kappa-light-chain-enhancer of activated B cells; *Cox-2*, cyclooxygenase-2; *inos*, inducible nitric oxide synthase; *Tnf-α*, tumor necrosis factor alpha; Il, interleukin; *Mcp-1*, monocyte chemoattractant protein-1; *PPAR-γ*, peroxisome proliferator-activated receptor gamma; *Sirt1*, sirtuin 1; *Ocln*, Occludin *Zo-1*, zonula occludens-1; *Cldn1*, Claudin1; *Muc*, mucin; *αsma*, alpha smooth muscle actin; *Ctgf*, connective tissue growth factor; *Col1a1*, collagen type I alpha 1 chain; *Gapdh*, Glyceraldehyde-3-phosphate dehydrogenase.

Table S3. List of antibodies for western blot analysis

Type	Antigen	Origin	Dilutions	Catalog number	Manufacturer
Primary antibody	pp65	Rabbit	1:1000	3033	Cell Signaling Technology
	p65	Rabbit	1:1000	8242	
	iNOS	Rabbit	1:1000	13120	
	COX-2	Rabbit	1:1000	12282	
	β-actin	Mouse	1:1000	12262	
Secondary antibody	Anti-rabbit IgG HRP-linked	Goat	1:3000	7074	
	Anti-mouse IgG HRP-linked	Horse	1:3000	7076	

pp65, Phospho-NF-kappaB p65; p65, NF-kappaB p65; iNOS, inducible nitric oxide synthase; COX-2, cyclooxygenase-2; IgG, Immunoglobulin G; HRP, horseradish peroxidase.

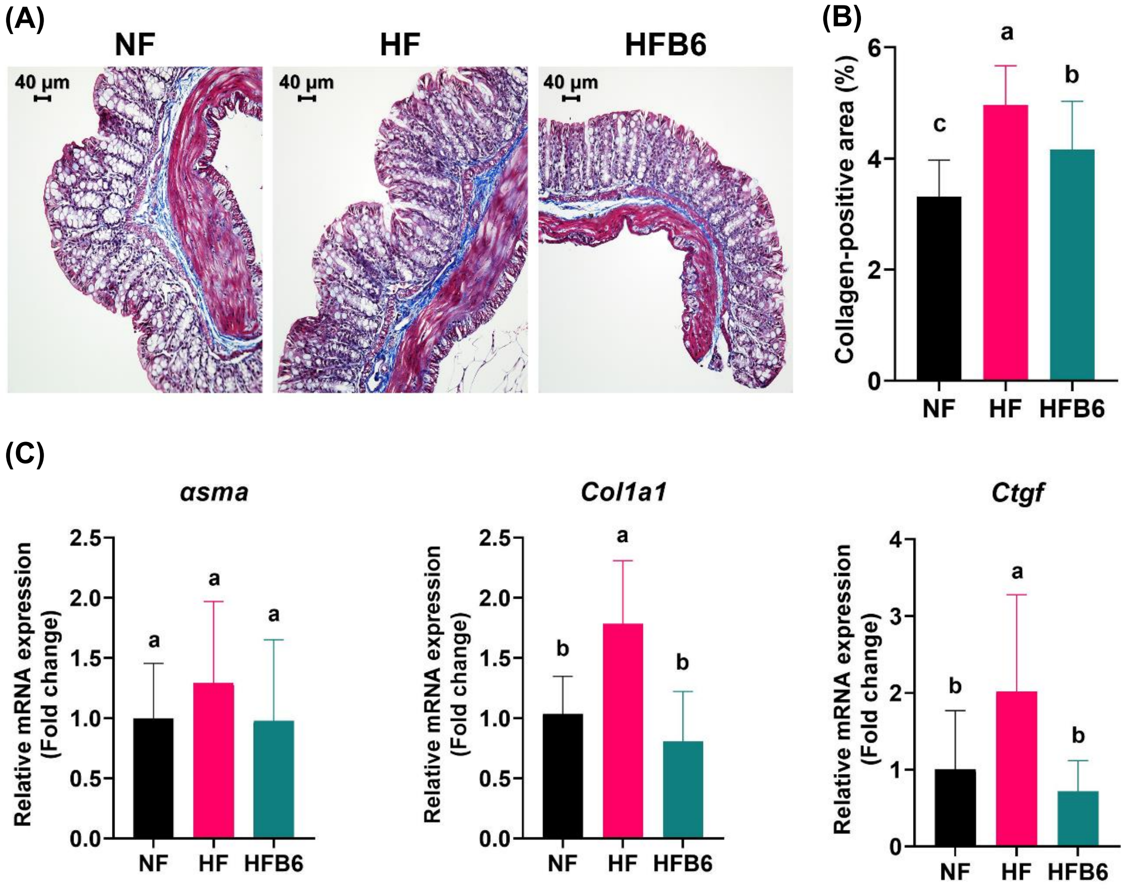

Figure S1. Effects of dietary intake of whole blackcurrant on the fibrosis of colonic tissue in high-fat diet-induced obese mice. **(A-B)** Representative microscopic images (×200; scale bars, 40 μm) and collagen-positive area of Masson's staining of the colon. Collagen deposits were dyed blue. The result was expressed as the mean ± standard deviation (SD) by evaluating the histological score in 5 fields of the stained slides of the colon in 4 animals per group. **(C)** Levels of gene expression of fibrosis-related factors in the colon. Data were expressed as mean ± SD of 8 animals per group. Significant differences assessed by one-way ANOVA and Tukey's post hoc tests were indicated by different superscripts (a, b, c) above the error bars (p < 0.05). NF, Normal diet group; HF, High-fat diet group; HFB6, High-fat diet group with 6% whole blackcurrant powder. *αsma*, alpha-smooth muscle actin; *Col1a1*, collagen type 1; *Ctgf*, connective tissue growth factor.
